# Combining the Strengths of the Explainable Boosting Machine and Metabolomics Approaches for Biomarker Discovery in Acute Myocardial Infarction

**DOI:** 10.3390/diagnostics14131353

**Published:** 2024-06-26

**Authors:** Ahmet Kadir Arslan, Fatma Hilal Yagin, Abdulmohsen Algarni, Fahaid AL-Hashem, Luca Paolo Ardigò

**Affiliations:** 1 Department of Biostatistics and Medical Informatics, Faculty of Medicine, Inonu University, Malatya 44280, Türkiye; arslan.ahmet@inonu.edu.tr; 2 Department of Computer Science, King Khalid University, Abha 61421, Saudi Arabia; 3 Department of Physiology, College of Medicine, King Khalid University, Abha 61421, Saudi Arabia; 4 Department of Teacher Education, NLA University College, 0166 Oslo, Norway

**Keywords:** Explainable Boosting Machine, acute myocardial infarction, metabolomics, biomarkers

## Abstract

Acute Myocardial Infarction (AMI), a common disease that can have serious consequences, occurs when myocardial blood flow stops due to occlusion of the coronary artery. Early and accurate prediction of AMI is critical for rapid prognosis and improved patient outcomes. Metabolomics, the study of small molecules within biological systems, is an effective tool used to discover biomarkers associated with many diseases. This study intended to construct a predictive model for AMI utilizing metabolomics data and an explainable machine learning approach called Explainable Boosting Machines (EBM). The EBM model was trained on a dataset of 102 prognostic metabolites gathered from 99 individuals, including 34 healthy controls and 65 AMI patients. After a comprehensive data preprocessing, 21 metabolites were determined as the candidate predictors to predict AMI. The EBM model displayed satisfactory performance in predicting AMI, with various classification performance metrics. The model’s predictions were based on the combined effects of individual metabolites and their interactions. In this context, the results obtained in two different EBM modeling, including both only individual metabolite features and their interaction effects, were discussed. The most important predictors included creatinine, nicotinamide, and isocitrate. These metabolites are involved in different biological activities, such as energy metabolism, DNA repair, and cellular signaling. The results demonstrate the potential of the combination of metabolomics and the EBM model in constructing reliable and interpretable prediction outputs for AMI. The discussed metabolite biomarkers may assist in early diagnosis, risk assessment, and personalized treatment methods for AMI patients. This study successfully developed a pipeline incorporating extensive data preprocessing and the EBM model to identify potential metabolite biomarkers for predicting AMI. The EBM model, with its ability to incorporate interaction terms, demonstrated satisfactory classification performance and revealed significant metabolite interactions that could be valuable in assessing AMI risk. However, the results obtained from this study should be validated with studies to be carried out in larger and well-defined samples.

## 1. Introduction

Acute myocardial infarction (AMI) stands as a critical juncture in cardiovascular health, characterized by the abrupt interruption of blood flow within the heart muscle [1]. This condition demands swift and precise intervention to avert severe consequences [2]. Comprising both ST-segment elevation myocardial infarction (STEMI) and non-ST-segment elevation myocardial infarction (NSTEMI), AMI subjects individuals to a heightened susceptibility to recurrent ischemic events [3]. The urgency of preventing AMI requires a comprehensive investigation of the complex pathways and contributing factors that lead to the risk.

In recent years, scientific studies have been carried out with “omics” approaches to identify biomarkers for various diseases. Metabolomics, one of these important approaches, involves the comprehensive analysis of small molecules called metabolites in cells, biological fluids, tissues, or organisms. Possible biomarkers to be determined by metabolomic analyses can be used in early diagnosis and follow-up of diseases and guidance of treatment strategies. Metabolomics is an area of omics sciences that focuses on the exhaustive study of small compounds or metabolites inside biological systems. The metabolites comprise diverse components such as carbohydrates, amino acids, lipids, and other chemical molecules. Metabolomics strives to comprehend the complex metabolic processes that occur within organisms and provides insights into their health, physiology, and responses to environmental factors [4,5].

Explainable Boosting Machines (EBM) is a sophisticated machine learning method that puts together the utilities of both boosting and interpretability. EBM is an interpretable machine learning model aimed to ensure both accurate predictions and insights into the model’s decision-making process and is particularly beneficial in cases where model interpretability is critical, such as credit scoring and healthcare. EBM is built on the concept of boosting, where numerous weak models are joined to form a powerful and useful predictive model. It consecutively fits a sequence of decision trees/weak learners, each one dedicated to correcting the errors committed by the former models [6,7].

The main objective of this study is to discover prognostic metabolite features that are candidates for predicting AMI using EBM. EBM was chosen in this research due to its speed and interpretability/explainability at local and global levels, as well as its advanced variable engineering features such as automatic binary interaction term detection. The secondary aim of the study is to investigate the contribution of binary interactions of metabolite biomarkers to the AMI classification performance and explainability of the model through automatic binary interaction term detection. For these purposes, a combined prognostic prediction model with the biomarker discovery process for AMI was developed. This framework enhances the precision of prognostic predictions for AMI and provides intuitive insights into these predictions through the consideration of metabolomics risk factors.

## 2. Materials and Methods

In this study, by following the MINIMAR (MINimum Information for Medical AI Reporting) [8], minimum reporting standards were tried to be ensured, especially in the model architecture and interpretation/evaluation of the results. The summary of both the coverage status of the MINIMAR and the key points of the Material and Methods section was provided in Supplementary Table S1.

### 2.1. The Data Set

This data is available at the NIH Common Fund’s National Metabolomics Data Repository (NMDR) website, the Metabolomics Workbench, https://www.metabolomicsworkbench.org (access date: 30 February 2024) where it has been assigned Project ID (ST001908) [9]. The dataset contains intensity values of 102 prognostic metabolites as continuous numeric type obtained by LC–MS (Liquid chromatography–mass spectrometry) technique and a binary outcome variable (Control/AMI). The list of 102 metabolites analyzed in the study and 21 metabolites included in the model training after the feature selection phase was provided in Supplementary Table S2. The current dataset consisted of 99 individuals, 34 healthy controls, and 65 individuals diagnosed with AMI. The gender distribution of the dataset is male (74, 74.7%) and female (25, 25.3%). According to the metadata in the Metabolomics Workbench database from which the dataset was downloaded, the study consisted of 100 individuals, but the downloaded dataset contained mass intensity values for 99 individuals.

### 2.2. Superficial Data Set Quality Check

The metabolite features were examined in terms of missingness and measures of dispersion by standard deviation, min–max scaling, and near-zero variance. No problems were encountered in the checks that could affect the next stages of data analysis.

#### 2.2.1. Outlier Analysis Phase

In this study, the OutlierTree [10] Python library (also the name of the method) was applied to carry out the outlier detection process. This method that leverages decision trees offers researchers an explainable perspective on outlier detection. To discover outliers in a dataset by constructing a tree-based model specifically optimized for outlier detection is aimed. This method identifies outliers without assigning scores to individual observations. For this, it builds decision trees to understand how different columns in the data relate to each other. It then examines these relationships to determine observations that stand out as significantly different from the rest [10,11].

#### 2.2.2. Missing Value Imputation Phase

To handle missing values, miceforest [12] Python library was employed. This tool aims to impute missing values using the LightGBM [13] model-based Multiple Imputation by Chained Equations (MICE) [14] approach. Using the MICE technique combined with the power of LightGBM, this approach provides computational speed, memory efficiency, and data type flexibility for missing value imputations [12].

#### 2.2.3. Feature Selection (FS) Phase

In this study, the Boruta method [15] was carried out for the FS task. Boruta is a robust and data-driven FS technique that can help researchers identify the most relevant predictors in their datasets. It is particularly beneficial in circumstances where it is wished to automate the FS process and avoid manually establishing feature importance thresholds. This feature enabled the Boruta technique to be used in this study. 

#### 2.2.4. Model Training Phase

- The employed model in a nutshell

EBM, founded in InterpretML [16] Python package and from the interpretable machine learning model family, is a model based on generalized additive models [17] with binary interaction terms. 

EBM uses a set of interpretable predictors for each instance to generate predictions. Making the model more interpretable compared to sophisticated models like deep neural networks, predictors are split into bins. The primary strength of EBM is its transparency. It ensures explicit insights into how each feature affects the prediction of the model. To achieve this mission, EBM generates global and local explanations (overall predictor importance and how features affect individual predictions). EBM produces predictor importance scores, which express the impact of each predictor on the model’s predictions. These scores are used to rank and prioritize predictors based on their contributions. The complexity of the EBM can be regulated by adjusting the number of weak models (trees) in the ensemble. This can help trade-offs among interpretability and prediction strength [16].

For any *i*th data point in the dataset (*x_i_, y_i_*), the form of EBM is characterized as [16]:

$$g(E[y])=\beta_{0}+\sum f_{i}(x_{i})+\sum f_{i,j}(x_{i},x_{j}),$$

where *g*(.), *β*_0_, *f_i_*, and *f_i,j_* represent link function, intercept, shape/smooth functions, and the interaction effect between features $x_{i},\;\mathrm{and}\;x_{j},$ respectively. The functions are learned by EBM employing enhanced ensemble learning tools. EBM provides a rapid implementation of the GA2M (Generalized Additive Model with Pairwise Interactions) algorithm. The binary interaction terms in the formula are automatically added to the model using the FAST (Feature-based Algorithm for Subgroup Discovery) technique [18].

b. The rest of the model training details

Before model training, the data set was randomly split into two parts for validating the model: training (75%) and test (25%) sets. To determine the value ranges of the optimization parameters of the EBM model, a comprehensive literature review was made, and the ranges specified in Table 1 were used in the model training process. In an attempt to find optimal hyperparameters, the grid search technique was used together with the 5-fold cross-validation method. The detailed information about the hyperparameters experimented during the training process is presented in Table 1.

After determining the optimal values for the hyperparameters, the model was retrained using the relevant values, and the prediction process was completed on the test data set.

#### 2.2.5. Model Performance Evaluations

The binary classification performance of the EBM model was performed using accuracy, sensitivity, specificity, F_1_ measure, and area under the ROC (Receiver Operating Characteristics) curve. A graphical representation called the ROC curve is used to assess how well a binary classification model performs. At different threshold values, it compares the True Positive Rate (TPR) against the False Positive Rate (FPR). Additionally, 95% bootstrapped confidence intervals with 1000 repetitions were calculated.

## 3. Results

Table 2 presents the distribution statistics and *p*-values of AMI and Control groups in terms of gender, age, Body Mass Index (BMI), and smoking.

**Figure 1:**
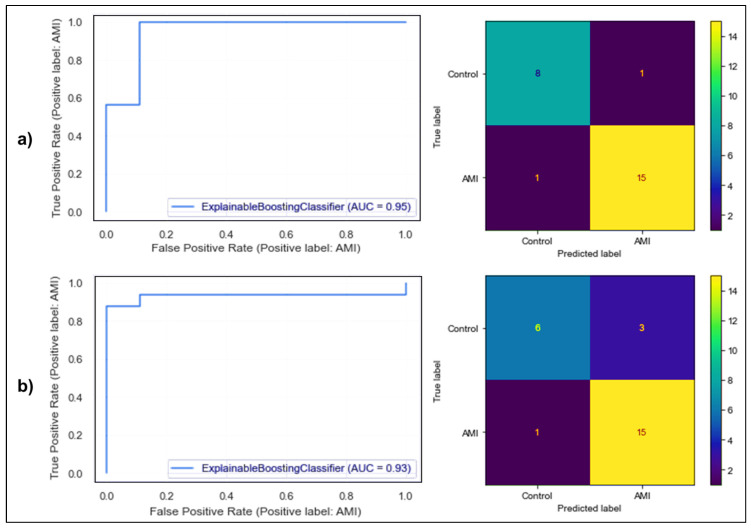
Confusion matrices and ROC curves for trained Explainable Boosting Machine Model Test Set predictions: There are two types of performance: (**a**) without interaction terms and (**b**) with interaction terms.

The superficial checks of the dataset showed there were no problems that could affect the data preprocessing and modeling steps. In the analyses conducted for outlier detection, a total of 13 suspected outliers were detected in different features. There were no missing values in the original data set. However, after the outlier analysis, 13 suspected outliers were removed from the dataset and replaced with “NA” (Not available). The missing values were imputed by the LightGBM-powered MICE method. In the feature selection analysis conducted after missing value imputation, the Boruta method selected 21 features out of 102 metabolite features, gender, age, body mass index (BMI), and smoking, and proceeded to the EBM modeling stage with these selected features. After parameter optimization, two EBM models were trained with and without the addition of interaction terms. The results of the confusion matrices and ROC plots of both models related to test classification performances are presented in Figure 1. Figure 1a shows that the EBM model with the interaction term misclassified only 2 instances and the area under the ROC curve of the model was 0.95. Figure 1b shows the performance outputs of the EBM model without the interaction term, which misclassified 4 instances and had an area under the ROC curve of 0.93. In addition, the measurements for both training and test classification performances of the related models by various metrics are given in Table 3 with 95% confidence intervals.

Upon evaluation of classification performances in Table 3, a notable sensitivity score was achieved in predicting AMI. A heightened sensitivity implies a reduced occurrence of false negatives (FN). In comparative biological studies, false positive and false negative errors are prevalent. Hence, establishing the probability of a genuine effect being significant holds paramount importance. A diminished FN value signifies a promising outcome for AMI instances. This outcome holds significant importance as the primary objective of this research is to minimize overlooked AMI cases (false negatives).

**Figure 2:**
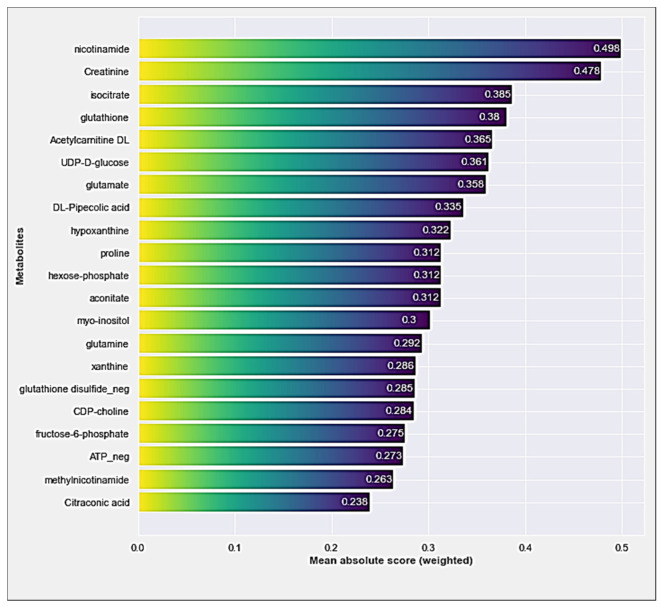
The feature importances of metabolites generated by the EBM model without adding interaction terms.

In Figure 2, the weighted mean absolute score-based feature importances for the two EBM models were demonstrated. The top three most important features determined by the EBM model without interaction terms were “Nicotinamide”, “Creatinine”, and “Isocitrate” (Figure 2). 

**Figure 3:**
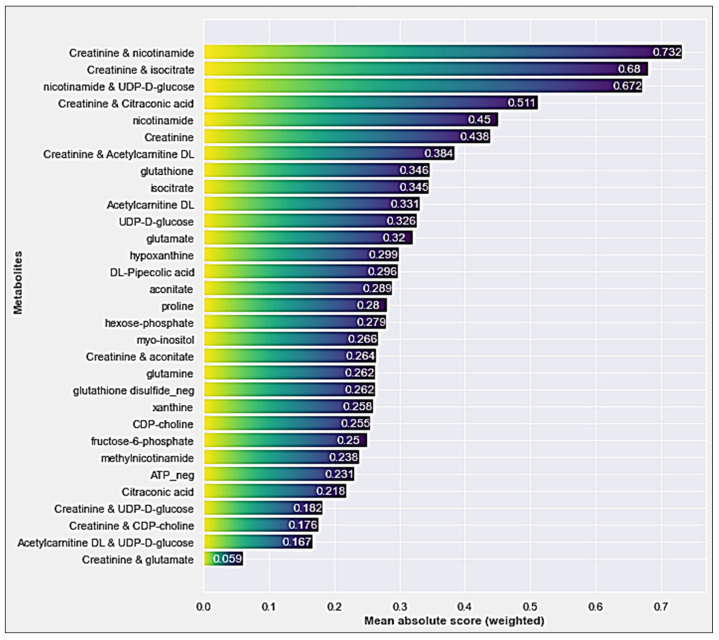
The feature importances of metabolites generated by the EBM model were trained by adding interaction terms.

Figure 3 shows the feature importance scores of the interaction terms added EBM model. When the feature importance outputs of the EBM model with interaction terms are examined, it is observed that the first 5 metabolites (Creatinine and nicotinamide, Creatinine and isocitrate, nicotinamide and UDP-D-glucose, Creatinine and Citraconic acid, nicotinamide) are mostly composed of the 3 metabolites mentioned above. 

**Figure 4:**
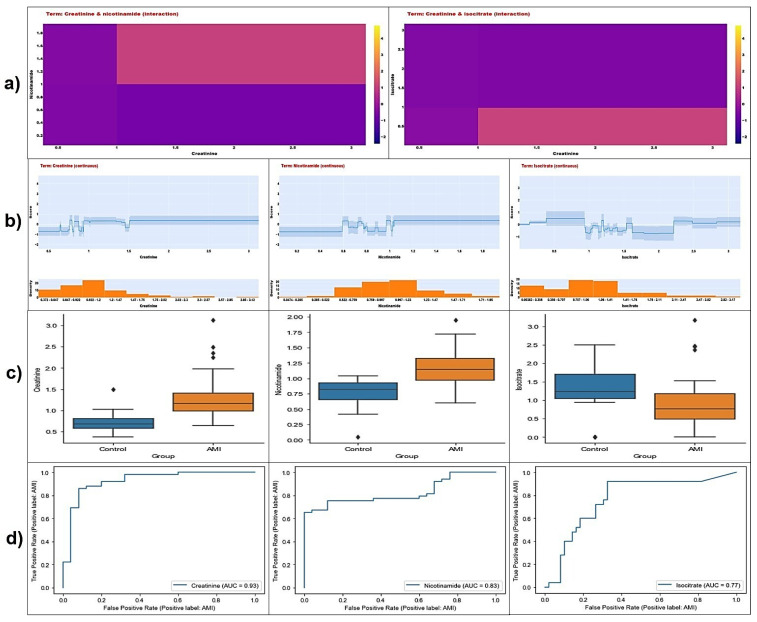
Interaction (**a**), distribution line (**b**), boxplot (**c**), and ROC (**d**) plots for the top 3 metabolites contributing the most to the model according to both the EBM models.

Figure 4 is shown divided into 4 rows. Row (a) shows the heat plots for the first two interaction terms with the highest weighted average absolute score according to the output of the EBM model with interaction terms. In this row, Nicotinamide x Creatinine and Isocitrate x Creatinine interactions are demonstrated, respectively. The pink-colored rectangles represent areas of increased risk of AMI. Row (b) shows the distribution of single-term metabolites for the same model and the effect of intensity variation on classification prediction and AMI risk. In the related graphs in Row (b), it can be said that the increase in creatinine and nicotinamide concentrations increases the risk of AMI. On the other hand, an increase in isocitrate concentration decreases the risk of AMI. In rows (c) and (d), boxplots and ROC plots from training data for creatinine, nicotinamide, and isocitrate metabolites are presented. Row (c) indicates that creatinine and nicotinamide concentration medians were higher and the isocitrate concentration median was lower in the AMI group. Row (d) shows that the creatinine metabolite feature has the highest ROC AUC value.

**Figure 5:**
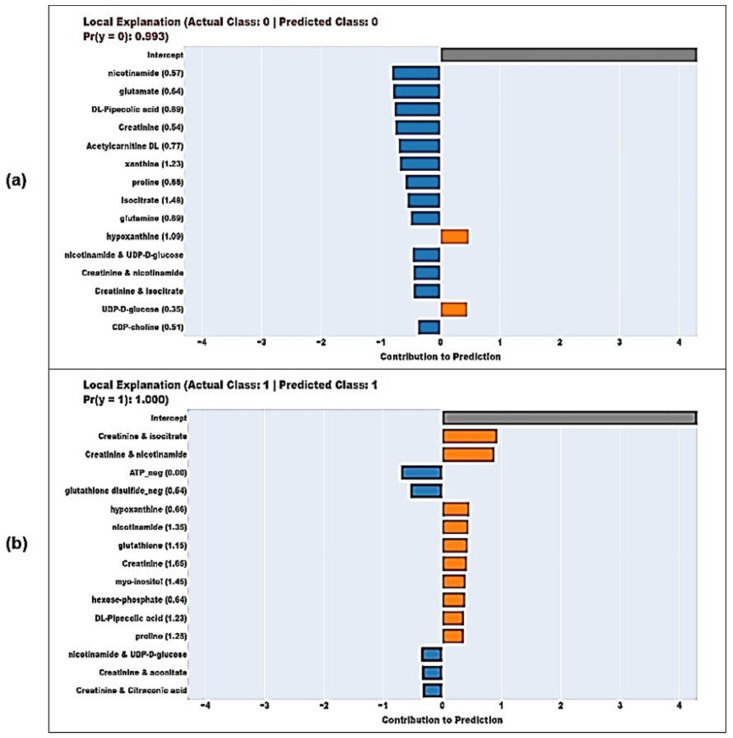
The local explanations of two samples that have the highest-class probabilities in terms of the Control (**a**) and AMI (**b**) groups.

In Figure 5, the local explanations of two samples having the highest-class probabilities in terms of the true predicted Control (a) and AMI (b) groups. The orange bars indicate the terms, contributing to the AMI classification and the blue bars indicate the terms contributing to the control group classification. For the sample labeled “Control” by the model, nicotinamide is the term that contributes most to the prediction. Also, for the sample labeled as “AMI” by the model, the feature that contributes the most to the prediction is the creatinine and isocitrate interaction term. For the relevant sample, the interaction terms, both involving the creatinine metabolite, are contributing to the model’s prediction of AMI.

## 4. Discussion

In this study, a pipeline including extensive data preprocessing stages and EBM, an explainable machine learning approach, was constructed to identify potential metabolite biomarkers that can be used to predict AMI. Additionally, the classification performance of the EBM model on the preprocessed training data was compared with and without adding interaction terms.

A study [19], which produced the dataset used in this study, aimed to find even more in-depth and interesting patterns for predicting AMI using various omics approaches than single omics studies. In this study, Random Forest was used as the classification model, 27 metabolite features were included in the modeling and the classification performance of the model was found to be 0.836 in terms of AUC ROC. In this study, the ROC AUC values of the EBM models trained in two different scenarios with 21 metabolite features were 0.93 and 0.95. Although the data sets considered are the same, it would not be fair to compare the two models as they are not subjected to the same data preprocessing process. However, it can be argued that EBM is more convenient than Random Forest for relevant tasks in terms of being boosting-based, detecting binary interaction terms automatically, and producing locally/globally interpretable results.

In another LC-MS/MS-based metabolomics research [20], unstable angina and AMI, subtypes of acute coronary syndrome (ACS), and healthy control groups were extensively compared in terms of serum and urine metabolites. In this multi-classification task performing Multinominal Adaptive LASSO and Random Forest models, 2-ketobutyric acid, LysoPC (18:2(9Z,12Z)), argininosuccinic acid, and cyclic GMP metabolites were identified as possible biomarkers for ACS prediction.

It is observed in Table 3 that the interaction term added EBM model outperforms the single term EBM model in terms of all classification performance metrics. The inclusion of automatically detected binary interaction terms in the EBM model is intended to improve model performance at the same time without compromising explainability, as stated by the developers of the model [14]. This is important because, in general, model prediction performance and explainability/interpretability are assumed as negatively correlated [21]. It can be stated that this is also confirmed in the current study when the classification metrics and unique explainable items produced by the models are considered.

When the feature importance plots obtained from both EBM models are examined, it is pointed out that the single and interaction effects of creatinine, nicotinamide, and isocitrate metabolites stand out in terms of contribution to the AMI classification performance. The discussion of the results will be based on these 3 metabolites.

Considering Figure 2 and Figure 3 together, it is observed that nicotinamide is the metabolite with the highest feature importance in EBM modeling without adding the interaction terms. In the EBM model with added interaction terms, it was observed that the metabolite with the fourth-highest feature importance (highest among single effects). Furthermore, the AUC value (0.83) obtained from the model-independent ROC analysis for nicotinamide has a good degree of discrimination (Figure 4c). Nicotinamide, also known as niacinamide, is a form of vitamin B3 and a versatile compound with many important functions such as energy metabolism, DNA repair, cellular signaling, and gene expression in the body [22,23,24,25]. Nicotinamide is a precursor to nicotinamide adenine dinucleotide (NAD+), a crucial coenzyme involved in various cellular processes, including energy production, DNA repair, and cell signaling. During AMI, the heart muscle experiences significant energy depletion. Nicotinamide’s potential to boost NAD+ levels could play a role in restoring energy balance and mitigating cellular damage. Nicotinamide exhibits antioxidant properties that might protect cardiac cells from oxidative stress, a major contributor to tissue damage during AMI [26,27,28].

In addition, the relationship between nicotinamide metabolite and the smoking status of the participants in this study was also examined. A total of 54 individuals in the dataset were current smokers, while the remaining 45 were either never smokers or former smokers. When these two groups were compared in terms of normalized nicotinamide concentration, a statistically significant difference was found between the groups (*p* = 0.002, *t* = 3.2, *df* = 97). Furthermore, the mean normalized nicotinamide concentrations were lower in the smoker group (Figure 4c). Also, when the percentages were analyzed, it was observed that the percentage of smokers in the AMI group (40.7%) was lower than in the Control group (59.3%) (Table 1). This is confirmed by the information that nicotinamide concentration is lower in smokers [29]. Perhaps this may suggest whether smoking status has a confounding effect on the determination of nicotinamide as a biomarker.

According to the results, the creatinine metabolite has the second highest feature importance in the single-effect EBM model. If the model with interaction terms is considered, the creatinine effect is present in 3 of the first 4 interaction terms that contribute the most to the classification performance. Similar to nicotinamide, it is observed that creatinine levels above certain normalized intensity values may increase the risk of AMI (Figure 4a,b). In addition, when the ROC analysis outputs were analyzed, the metabolite with the highest AUC value among the 3 metabolites was creatinine Figure 4b. The boxplots show that the median of nicotinamide and creatinine mass density values were higher in the AMI group (Figure 4c).

Creatinine is a waste product from the breakdown of creatinine phosphate, an amino acid that muscles use to produce energy [30]. Elevated levels of AMI may indicate damage to the heart muscle as a direct consequence of the infarction (heart attack). Creatinine is also an important indicator of kidney function. During AMI, decreased blood flow to the kidneys can impair their function, leading to reduced creatinine clearance and subsequently elevated serum levels. Creatine, the precursor to creatinine, plays a vital role in cellular energy metabolism. Disrupted energy production during AMI could contribute to alterations in creatinine levels [31,32,33]. The studies investigating the utility of creatinine as a risk factor for heart disease have shown that people with high levels of creatinine metabolites have a higher risk of developing heart disease [34,35]. In the case of AMI, an observational study in 2019 investigated the metabolic features of CHD patients using a targeted metabolomics approach. Blood samples were collected from 302 patients with CHD and 59 normal coronary artery (NCA) subjects and analyzed using the LC-MS technique. According to the results, it was determined that creatinine concentration was significantly higher in the CHD group than in the NCA group, including patients with AMI [36].

Furthermore, in the related study [37] that produced the dataset used in this study, both creatinine and nicotinamide metabolite levels were found to be up-regulated. This indicates that the analyte concentrations of these two biomarkers are higher in patients with AMI than in the control group.

In this study, isocitrate was observed as the 3rd most important metabolite feature. Isocitrate is a molecule involved in a series of chemical reactions known as the Krebs cycle (TCA cycle or citric acid cycle). The Krebs cycle is a series of reactions that cells use to produce energy [38,39]. Isocitrate is an intermediate in the tricarboxylic acid (TCA) cycle, the central pathway for cellular energy production. Disruptions to the TCA cycle during AMI can impact isocitrate levels. Elevated isocitrate might indicate impaired energy metabolism in the heart muscle. The TCA cycle occurs within mitochondria, the cellular powerhouses. Alterations in isocitrate levels could reflect mitochondrial dysfunction, a hallmark of AMI [40,41].

However, the study [37] that provided the data set used in the current study found that the relevant metabolite was down-regulated in AMI patients compared to the control group, indicating a lower intensity of the analyte. This output is in line with the results of the current study.

## 5. Conclusions

In this study, to determine the effect of potential biomarkers that can be evaluated to predict AMI and their interactions on AMI prediction was aimed. For this purpose, two different variants of the EBM model were trained on the training dataset and these two cases were compared with each other using various classification metrics and feature importance values. The EBM model with interaction terms provided satisfactory classification performance and identified metabolite interactions that can be considered in assessing the risk of AMI. Furthermore, the fact that it contributes to the examination of the metabolite contribution of AMI risk on a sample-based and that it generates these findings on its own and not using external model agnostic methods such as SHAP (Shapley Additive explanations), etc., makes the EBM model attractive for use as an explainable/interpretable model in metabolomics research. In conclusion, this study successfully developed a pipeline incorporating extensive data preprocessing and the EBM model to identify potential metabolite biomarkers for predicting AMI. The EBM model, with its ability to incorporate interaction terms, demonstrated satisfactory classification performance and revealed significant metabolite interactions that could be valuable in assessing AMI risk.

## 6. Limitations and Future Works

The two main limitations of this study are the relatively small sample size and the lack of an external validation process that would enable a more robust assessment of the classification performance and other outputs of the model. The application of the model in a multi-omics study in combination with other omics approaches to identify AMI risk factors and determine their contribution more robustly is suggested as future research.

## Figures and Tables

**Table 1:** Detailed information about the optimal hyperparameters.

Hyperparameters	Candidate Value	Determined Value
“Outer bags”	1 to 11; step = 1	10
“Learning rate”	[0.001, 0.005, 0.01]	0.01
“Early stopping rounds”	35 to 41; step = 1	37
“Max rounds”	9000 to 10,000; step = 100	10,000
“Max leaves”	5 to 11; step = 1	10

**Table 2:** The descriptive and inferential statistics of AMI and Control groups in terms of some factors.

Variables	Groups		*p*
	AMI	Control	
	(n = 65)	(n = 34)	
Gender			**<0.001**
Female	4 (16%)	21 (84%)	
Male	61 (82.4%)	13 (17.6%)	
Age	55.72 ± 9.01	56.35 ± 8.86	0.74
BMI	25.26 ± 3.74	25.07 ± 3.86	0.82
Smoking			
Yes	22 (40.7%)	32 (59.3%)	**<0.001**
No	43 (95.6%)	2 (4.4%)	

**Table 3:** The outputs show the binary classification performance of the EBM model with and without the addition of interaction terms.

Metric	Interaction Terms Added?	Data Source	Value	BCI * (95%)
**Accuracy**	Yes	Train	1.00	(0.99–1.00)
		**Test**	**0.92**	**(0.80–1.00)**
	No	Train	1.00	(0.99–1.00)
		Test	0.84	(0.68–0.96)
**Sensitivity**	Yes	Train	1.00	(0.99–1.00)
		**Test**	**0.89**	**(0.67–1.00)**
	No	Train	1.00	(0.99–1.00)
		Test	0.83	(0.65–1.00)
**Specificity**	Yes	Train	1.00	(0.99–1.00)
		**Test**	**0.94**	**(0.80–1.00)**
	No	Train	1.00	(0.99–1.00)
		Test	0.86	(0.50–1.00)
**F_1_ score**	Yes	Train	1.00	(0.99–1.00)
		**Test**	**0.94**	**(0.83–1.00)**
	No	Train	1.00	(0.99–1.00)
		Test	0.88	(0.73–0.98)
**AUC**	Yes	Train	1.00	(0.99–1.00)
		**Test**	**0.95**	**(0.83–1.00)**
	No	Train	1.00	(0.99–1.00)
		Test	0.93	(0.79–1.00)

*: Bootstrapped confidence interval with 1000 repetitions.

## Data Availability

The data is open access and can be requested from the corresponding author upon appropriate request.

## Supplementary Materials

The following supporting information can be downloaded at: https://www.mdpi.com/article/10.3390/diagnostics14131353/s1, Table S1: Highlights for Material and Methods and coverage status of the MINIMAR; Table S2: The list of 102 metabolites analyzed in the study and 21 metabolites included in the model training after the feature selection phase.
