# Supplementary material for: Combining the Strengths of the Explainable Boosting Machine and Metabolomics Approaches for Biomarker Discovery in Acute Myocardial Infarction
Source: Diagnostics (Basel). 2024 Jun 26;14(13):1353. doi: 10.3390/diagnostics14131353 (PMC11240568; doi:10.3390/diagnostics14131353)
Supplement: Supplementary file 1 [file diagnostics-14-01353-s001.zip › diagnostics-3057110-supplementary.pdf]

Supplementary Table S1: Highlights for Material and Methods and coverage status of the MINIMAR

Component	Description	Coverage status	Additional info
**Study population and setting**	Population	✓	Provided on the study page*
	Study setting	✓	Provided on the study page*
	Data source	✓	Retrieved from Metabolomics Workbench/National Metabolomics Data Repository (NMDR) (https://www.metabolomicsworkbench.org) (Study ID: ST001908)
	Cohort selection	✓	Inclusion and exclusion criteria were provided in the study page*
**Patient demographic characteristics**	Age	✓	The mean age for the Control group: was 56.35<br>The mean age for the AMI group was 55.72
	Sex	✓	Gender distribution: male (74, 74.7%), female (25, 25.3%)
	Race	✓	Asian
	Ethnicity	✓	Chinese, Indian, Malay
	Socioeconomic status	X	Not provided
**Model architecture**	Model output	✓	Evaluation and interpretation outputs were specified in the Material and Methods section.
	Target user	✓	Clinicians, data scientist
	Data splitting	✓	Simple random split validation (75% - 25%) with 5-folds cross-validation (on training data)
	Gold standard	✓	Binary outcome variable (Control/AMI)
	Model task	✓	Binary classification
	Model architecture	✓	Explainable Boosting Machine (EBM)
	Features	✓	Contains 102 prognostic metabolites as continuous numeric type, gender, age, body mass index (BMI), smoking, and a binary outcome variable (Control/AMI). Median normalization was also applied to prognostic features.
	Missingness	✓	There are no missing values in the original data set, but the missing values that occurred after the outlier analysis were imputed by the LightGBM model-based Multiple Imputation by Chained Equations (MICE) approach.
**Model evaluation**	Optimization	✓	Grid search with 10800 iterations (for 5 hyperparameters)
	Internal model validation	✓	Simple random split validation (75% - 25%) with 5-folds cross-validation (on training data)
	External model validation	X	Not provided
	Transparency	✓	The dataset is available to researchers at the link*.<br>The Python code pipeline containing the analyses conducted in the study is shared on the GitHub page**.

*: http://dx.doi.org/10.21228/M8BD7S
**: https://github.com/akarslan/biostatapps/tree/master/EBM_AMI

Supplementary Table S2: The list of 102 metabolites analyzed in the study and 21 metabolites included in the model training after the feature selection phase

Metabolites	Included after FS?
1-Methyladenosine	No
2-Aminooctanoic acid	No
2-Hydroxy-2-methylbutanedioic acid	No
2-hydroxygluterate	No
2-Isopropylmalic acid	No
Acetylcarnitine DL	Yes
aconitate	Yes
adenine	No
adenosine	No
ADP_neg	No
a-ketoglutarate	No
alanine	No
AMP	No
anthranilate	No
arginine	No
Ascorbic acid	No
asparagine	No
aspartate	No
ATP_neg	Yes
betaine	No
Carbamoyl phosphate	No
carnitine	No
CDP-choline	Yes
CDP-ethanolamine	No
cholesteryl sulfate	No
choline	No
Citraconic acid	Yes
citrulline	No
CMP	No
Creatinine	Yes
cyclic-AMP	No
cytidine	No
cytosine	No
deoxyadenosine	No
deoxyinosine	No
dGDP_neg	No
D-glucarate	No
D-gluconate	No
D-glyceraldehdye-3-phosphate	No
dihydroxy-acetone-phosphate	No
dimethylglycine	No
DL-Pipecolic acid	Yes
D-sedoheptulose-1-7-phosphate	No
FAD	No
fructose-6-phosphate	Yes
glucose-1-phosphate	No
glutamate	Yes
glutamine	Yes
glutathione	Yes
glutathione disulfide_neg	Yes
glutathione disulfide_pos	No
glutathione_neg	No
Glycerophosphocholine	No
GMP	No
hexose-phosphate	Yes
histidine	No
hydroxyphenylpyruvate	No
hypoxanthine	Yes
IMP	No
inosine	No
isocitrate	Yes
lactate	No
leucine-isoleucine	No
lysine	No
malate	No
methionine	No
Methylcysteine	No
methylnicotinamide	Yes
myo-inositol	Yes
N-acetyl-glucosamine-1-phosphate	No
NAD+_pos	No
Ng,NG-dimethyl-L-arginine	No
nicotinamide	Yes
nicotinate	No
ornithine	No
orotate	No
phenylalanine	No
Phenylpropiolic acid	No
Phosphorylcholine	No
p-hydroxybenzoate	No
proline	Yes
ribose-phosphate	No
S-adenosyl-L-homoCysteine_pos	No
sarcosine	No
serine	No
shikimate	No
S-methyl-5-thioadenosine	No
S-ribosyl-L-homocysteine_neg	No

S-ribosyl-L-homocysteine_pos	No
succinate	No
taurine	No
threonine	No
tryptophan	No
tyrosine	No
UDP_neg	No
UDP-D-glucose	Yes
UDP-D-glucuronate	No
UDP-N-acetyl-glucosamine	No
UMP	No
Uric acid	No
valine	No
xanthine	Yes
